# Case report: Two cases of pyloric adenomas with high-grade intraepithelial neoplasia at distinct locations

**DOI:** 10.3389/fimmu.2024.1485840

**Published:** 2024-12-13

**Authors:** Yuetian Li, Shuai Chen, Xuechun Zhao, Quanyi Wang

**Affiliations:** ^1^ Clinical School of Medicine, Jining Medical University, Jining, Shandong, China; ^2^ Department of Pathology, Affiliated Hospital of Jining Medical University, Jining, Shandong, China

**Keywords:** pyloric adenoma, high-grade intraepithelial neoplasia, gastric adenoma, duodenum, immunohistochemistry

## Abstract

The rare gastrointestinal tract epithelial polyp known as a pyloric gland adenoma (PGA) is more common in elderly women and uncommon in the duodenum. There are reports of two PGA cases involving high-grade intraepithelial neoplasia. A 75-year-old man was admitted to the hospital as Patient 1 due to “epigastric distension and pain for more than 10 days”. The mucous membrane around the stomach fundus and body was thin, and an electron gastroscopy revealed a large, thick protrusion in the stomach fundus that measured about 6 cm in circumference. High-grade intraepithelial neoplasia and stomach pyloric adenoma are the pathological diagnoses. An outside hospital provided consultation for Patient 2, a 68-year-old male. Pyloric adenoma at duodenal bulb with high-grade intraepithelial neoplasia is the pathological diagnosis. The single layer of cuboidal to low columnar epithelial cells with rounded nuclei and eosinophilic cytoplasm was surrounded by densely packed glands with sporadic cystic dilatation that made up the tumor tissue. The diagnosis of PGA required the immunohistochemical labeling of MUC6. In both cases, simple surgical resection was carried out, and followed up for half a year, no recurrence.

## Introduction

PGA are uncommon mass-forming neoplasms that are comprised of densely packed pyloric glands (1). Chronic injury to the mucosa, particularly in the body of the stomach, which is covered by the mucosa of the fundic glands, is the most common cause of this condition. It can also occur in other regions of the duodenum, gallbladder, bile ducts, and esophagus (2). The condition appears most frequently in older women. PGA is a precancerous condition that is disseminated or observed in patients with genetic syndromes, including Lynch Syndrome, Familial Adenomatous Polyposis (FAP), and Juvenile Polyposis Syndrome (3). Additionally, a small number of cases have been reported in recent years of PGA in MUTYH-associated polyposis, an autosomal recessive FAP-reduced type (4). Sporadic gastric PGA is the result of mucosal degeneration caused by Helicobacter pylori infection or autoimmune gastritis (5). The genesis of sporadic PGA is significantly influenced by persistent inflammation-induced pseudopylorosis of the fundic glands. In contrast, FAP-associated PGA is primarily associated with fundic gland polyposis, occurs in the primitive fundic gland mucosa, and is more prevalent in younger patients without a gender difference.

## Case presentation

### Patient 1

Patient 1 is a 75-year-old male who was admitted to the Affiliated Hospital of Jining Medical University on November 28, 2023, due to epigastric distension and pain that had persisted for more than ten days. The patient was confined to Sishui County Rehabilitation Hospital on November 28, 2023, due to “epigastric distension and discomfort sustained for more than 10 days”. The patient experienced epigastric distension and discomfort for over 10 days without any discernible cause, particularly in the right upper abdomen. The discomfort was exacerbated by eating, and it was not alleviated by changing position. The patient did not experience radiating pain in the lower back, nausea, vomiting, bloody vomiting, black stools, or any other discomforts. He sought treatment at Sishui County Rehabilitation Hospital. Previous history: “arrhythmia,sinus bradycardia” for over six years,no history of alcohol consumption,have abstained from smoking for over six years,no family history of genetic diseases.

**Figure 1:**
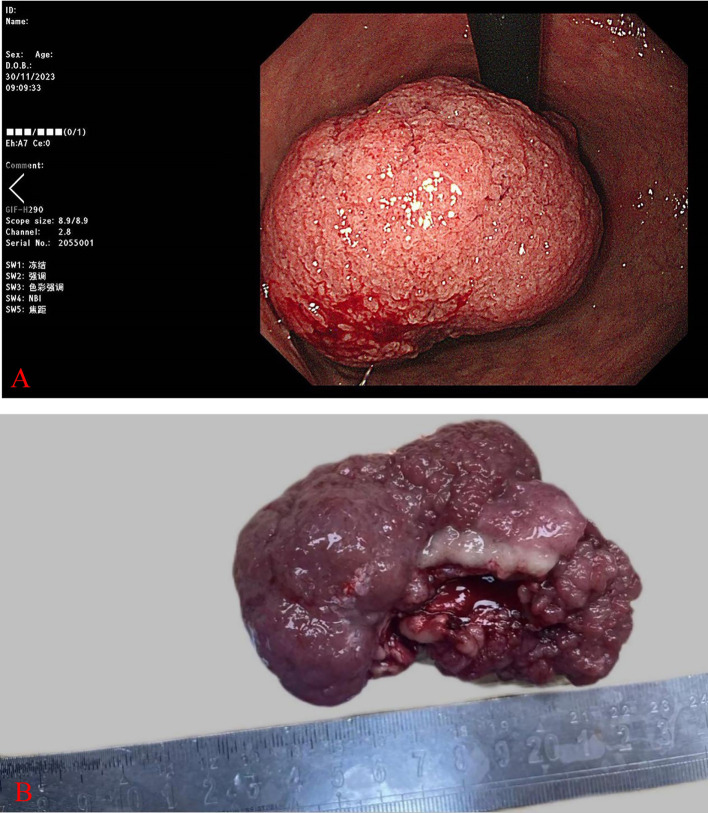
Patient 1 The e-gastroscopy revealed a prominent thick-tipped protrusion of approximately 6 cm in diameter in the gastric fundus, with a lobulated surface **(A)**. Patient 1 Gross presentation of the swelling **(B)**.

Specialization: abdomen that is flat, lacks gastrointestinal-type peristaltic waves, superficial abdominal wall varicose veins, spongy abdominal wall, right epigastric tenderness, no rebound tenderness, and no mass. The spleen was not palpated, there was no percussion pain in the hepatic region, mobile turbidities were negative, and gastrointestinal sounds were normal. Murphy’s sign was negative. The patient was admitted to the hospital in order to enhance the relevant examination. CT on November 29, 2023:1. neoplastic lesions can be considered in the cardia, gastric fundus, gastric sinus part, pylorus, duodenal bulb, and descending section of the wall that exhibit diffuse hypertrophy. It is advisable to combine this with the enhancement of CT examination. 2. Dilatation of the common bile duct, pancreatic duct, and intra- and extra-hepatic bile ducts 3. Cholecystitis, gallbladder stones, and gallbladder diverticulum are potential complications. Electron gastroscopy: the gastric fundus is a vast, thick tibial bulge with a diameter of approximately 6 cm. The surface is lobulated, and the gastric fundus is brittle and has poor elasticity (**Figure 1A**). The body mucosa is thin, and the submucosal vascular network is visible through the translucent mucus lake. The biopsy was performed in six pieces.

**Figure 2:**
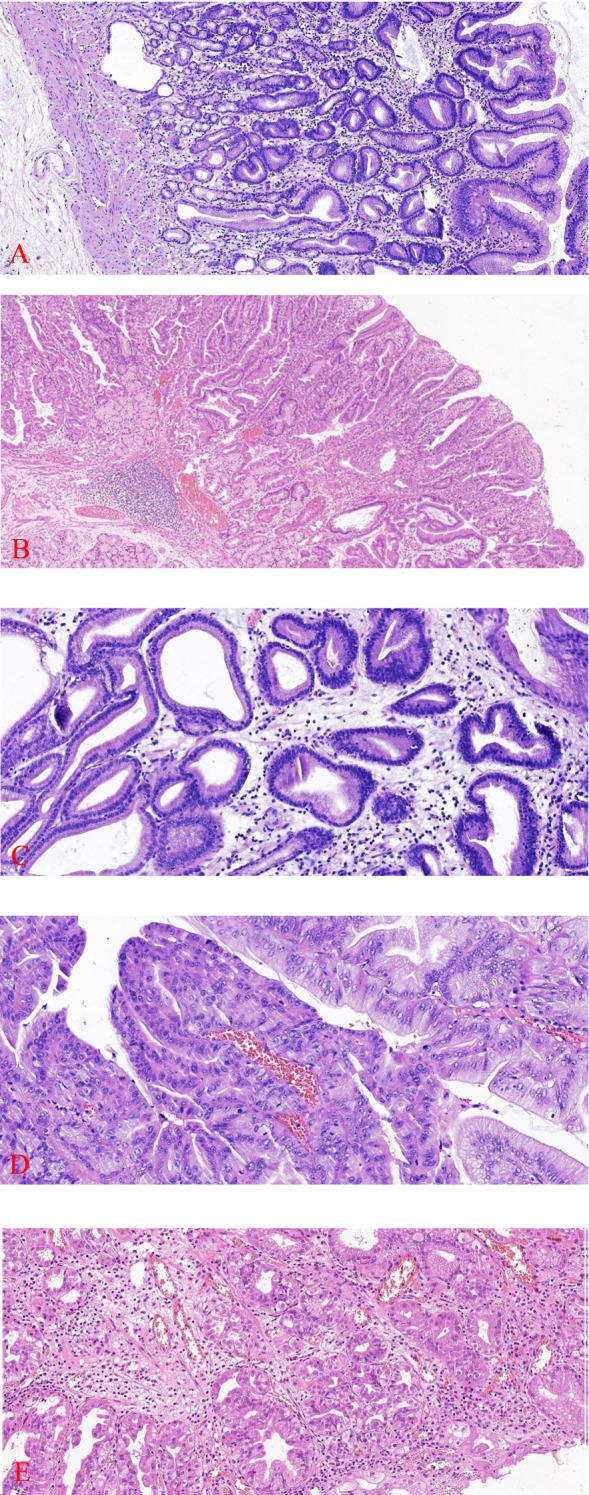
Tumor tissue from Patient 1 exhibits an elevated growth pattern, with varying degrees of contorted and dilated glands visible (HE×100) **(A)**. Patient 2 has tumor tissue that is restricted to superficial mucosal proliferation (HE×100) **(B)**. Patient 1 has tumor tissue that is composed of pyloric ductal glands (HE×200) **(C)**. Patient 1 exhibited focal papillary growth of tumor tissue with a visible fibrovascular axis (HE×200) **(D)**. Patient 2 was diagnosed with high-grade intraepithelial neoplasia, a form of focal glandular hyperplasia characterized by a dilated lumen, congested arrangement, enlarged nuclei, reduced cytoplasm, increased nucleoplasmic ratio, pronounced nucleoli, and loss of cellular polarity(HE ×200) **(E)**.

Pathological examination: The intraoperative cooling of Patient 1 revealed a single mass that measured 9×7×4cm and had a brittle, cauliflower-like cut surface. What was observed under a microscope: The tumor tissue in patient 1 was primarily cauliflower-like and had been sufficiently sampled, with no definite carcinoma present (**Figure 1B**). Patient 1 exhibited an elevated pattern of tumor tissue growing along the superficial mucosa, with cystically dilated glands of varying diameters and large, dark-stained, intricately arranged nuclei (**Figure 2A**). The tumor tissue in Patient 1 was composed of pyloric tubular ducts that were covered with cuboidal or columnar mucus-secreting cells that lacked apical mucin caps (**Figure 2C**). The tumor tissue of Patient 1 exhibited elongated gastric dimples, focal papillary growth, a visible fibrovascular axis, a loss of cell polarity, pronounced nucleoli, and significant heterogeneity (**Figure 2D**). Pathologic diagnosis: Patient 1 had a (gastric) pyloric adenoma with high-grade intraepithelial neoplasia. Immunohistochemistry: Ki-67 (+,5-8%), MUC-5AC (+).

### Patient 2

**Figure 3:**
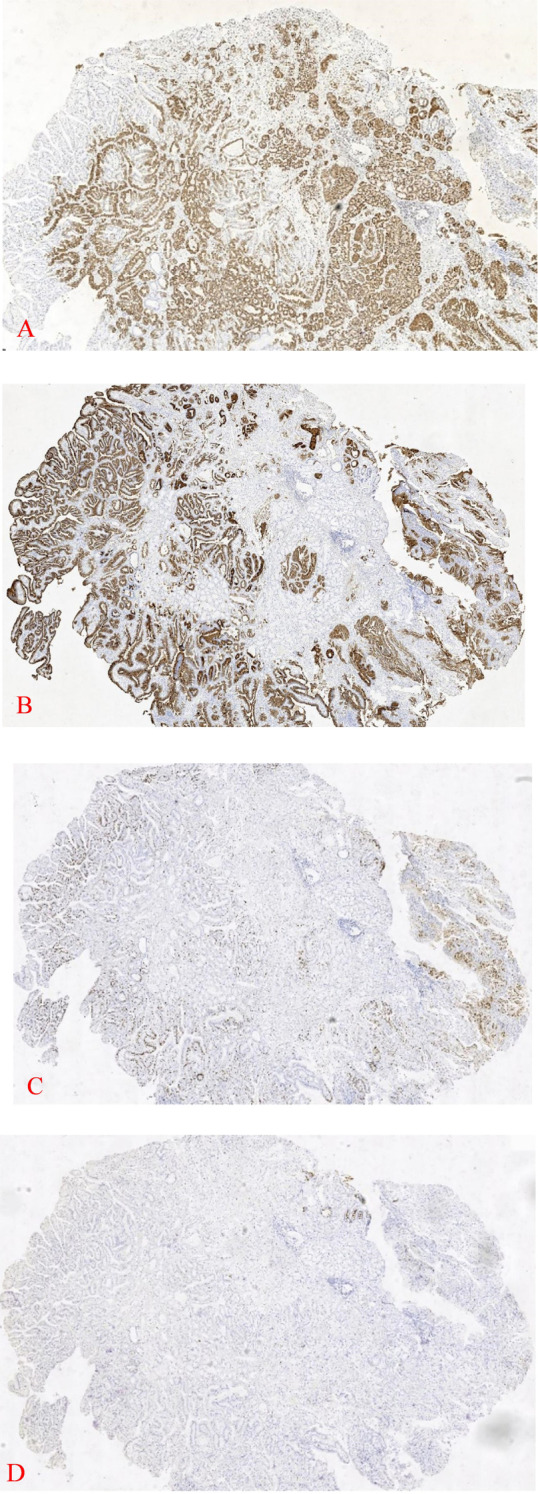
1 Patient 2 exhibits diffuse expression of MUC-6 in deep tissues (IHC×40) **(A)**. The tumor tissue surface of Patient 2 was positive for MUC-5AC (IHC×40) **(B)**. Tumor tissue from Patient 2 was Ki-67 positive (IHC×40) **(C)**. Tumor tissue from Patient 2 was found to be positive for CD10 (IHC×40) **(D)**.

In an outer hospital, patient 2, a 68-year-old male, was diagnosed with a duodenal bulb adenoma during a physical examination. In order to obtain additional diagnosis and treatment, he was transferred to our facility. Pathological examination:Patient 2 (duodenal bulb): A soft tissue with a maximal transverse diameter of 1.3 cm and a greyish red color. From a microscopic perspective: patient 2 The superficial mucosal layer was the sole location of the tumor tissue (**Figure 2B**). Patient 2’s tumor tissue was composed of a single layer of cuboidal to low columnar epithelial cells with round nuclei and eosinophilic cytoplasm. The diagnosis of high-grade intraepithelial neoplasia was made as a result of the tumor cells’ enlarged nuclei, crowded nuclei, increased nuclear schizophrenia, evident nucleoli, highly heterogeneous cells, loss of cell polarity, and disorganized arrangement (**Figure 2E**). Immunohistochemistry: MUC6 is expressed in deep pyloric glands, whereas MUC5AC is a surface gastric minor concave mucin.Tumor tissues exhibited diffusely positive expression of MUC-6 (**Figure 3A**). MUC-5AC was found to be positively expressed on the surface of the tumor tissues (**Figure 3B**). The tumor tissues exhibited a high level of expression of Ki-67 and P53 (+, mutant) (**Figure 3C**). CD10 exhibited dispersed positivity (**Figure 3D**). Two cases of pyloric adenomas with high-grade intraepithelial neoplasia at distinct locations). Pathologic diagnosis: Patient 2 demonstrated a high-grade intraepithelial neoplasia with a (duodenal bulb) pyloric adenoma. Immunohistochemistry: MUC-5AC (+), MUC-6 (+), MUC-2 partially positive, CD10 scattered positive, P53 (mutant), Ki-67 (+, 5-8%).

## Discussion

Endoscopically or morphologically, PGA typically manifests as a polypoid lesion; however, it may also manifest as an irregular, flattened lesion or mucosal ulcer. It is histologically similar to the gastric pyloric glands, with tightly packed glands (occasionally with cystic dilatation) and a single layer of cuboidal to low columnar epithelial cells and round to oval nuclei (6). The cytoplasm is light-stained to eosinophilic, ground-glass, and lacks mucus on the apical mucinous caplets (7).

Types of gastric adenomas include those that are gastric and intestinal (8). A rare gastric-type duodenal tumor, duodenal PGA is most frequently observed in elderly individuals and is caused by ectopic gastric mucosa (9). It is typically a polypoid located in the proximal duodenum (10). MUC6 is expressed in the deep pyloric glands, whereas MUC5AC is a surface gastric minor concave mucin. PGA exhibited diffuse MUC6 (+) and surface MUC5AC (+) as evidenced by immunohistochemistry. The pyloric gland adenoma is a precancerous lesion that has the potential to progress to low-grade intraepithelial neoplasia, high-grade intraepithelial neoplasia, and ultimately, adenocarcinoma (11). Tubular-chromaffin-like structures are the most common histological features of PGA, with very few chorionic structures. Severe heterogeneous hyperplasia and adenocarcinoma are associated with larger and tubular-chorionic structures (12).

The tumorigenicity of pyloric adenomas is indicated by the accumulation of *P53*, mutations in the oncogenes *GNAS, KRAS, CTTNB1*, oncogene *SMAD4*, and *TP53*, as indicated by molecular genetics results (13). MUC6 and TFF2 are secreted by pyloric gland cells, and they bind to each other through the α-1,4-linked αGlcNAc at the MUC6 terminus (14). The reduced expression of αGlcNAc and TFF2 indicates that PGA has malignant potential, as αGlcNAc is a gastric tumor suppressor.The differential diagnosis of PGA necessitates the distinction between tiny concave adenomas and intestinal-type adenomas. The gastric notochordal adenoma is characterized by a proliferating columnar notochordal epithelium with elongated nuclei, well-formed apical mucin caps, immunohistochemical markers MUC5AC (+) and MUC6 (-), rounded or ellipsoidal nuclei with an inconspicuous nucleolus, absence of apical mucin caps, and immunohistochemical markers MUC5AC and MUC6 (+) (15). Furthermore, the tumorigenic glands of PGA are dispersed throughout the lesion, in contrast to the gastric lesser concave type adenomas, which develop primarily on the surface. Intestinal-type adenomas are composed of pseudoproliferative cells that exhibit thrush cell or PAN cell differentiation and exhibit greater cellular heterogeneity. They typically exhibit diffuse staining for MUC2, CD10, and CDX2, as well as the absence of an apical mucin cap. However, PGA exhibits either no staining or only focal staining, which indicates a heterogeneous lesion phenotype. The presence of gastric-type mucin can confirm that PGA has gastric differentiation. Focal staining for MUC6 may be observed in certain intestinal-type adenomas, particularly those with tubular structures. Nevertheless, the lesions will not exhibit diffuse coloration, as is the case with PGA.

Tumor tissue must be fixed as soon as possible and adequately after ex vivo, and all of the tissue should be extracted in accordance with the standard criteria. If required, the films should be meticulously examined with the assistance of immunohistochemistry or specialized stains to facilitate the interpretation of the presence of high-grade or malignant lesions. PGAs are conservatively treated and have a low recurrence rate. However, complete resection by ESD is also feasible when they are large or exhibit high-grade features (16).

## Conclusion

In this paper, we present two cases of PGA in distinct regions of the body, both of which are older men. Patient 1 was admitted to the hospital due to cholecystitis attacks that resulted in abdominal pain. CT imaging suggests that the gastric fundus body has thickened, which was subsequently diagnosed as PGA. The two PGA patients in this paper have no apparent symptoms, and the physical examination of the lesion indicates that the onset of PGA is more insidious. In both cases, simple surgical resection was carried out, and followed up for half a year, no recurrence. We suggest that patients with this characteristic undergo a close follow-up (17). The risk of malignant transformation of PGA necessitates that we remain vigilant and strive to detect, diagnose, and treat it as early as possible (18).

## Data Availability

The original contributions presented in the study are included in the article/supplementary material. Further inquiries can be directed to the corresponding author.
